# Polarization vision mitigates visual noise from flickering light underwater

**DOI:** 10.1126/sciadv.abq2770

**Published:** 2022-09-09

**Authors:** Siân Vincent Venables, Christian Drerup, Samuel B. Powell, N. Justin Marshall, James E. Herbert-Read, Martin J. How

**Affiliations:** ^1^ School of Biological Sciences, University of Bristol, UK.; ^2^ Department of Zoology, University of Cambridge, UK.; ^3^ Queensland Brain Institute, University of Queensland, Australia.; ^4^ Aquatic Ecology Unit, Department of Biology, Lund University, Sweden.

## Abstract

In shallow water, downwelling light is refracted from surface waves onto the substrate creating bands of light that fluctuate in both time and space, known as caustics. This dynamic illumination can be a visual hindrance for animals in shallow underwater environments. Animals in such habitats may have evolved to use polarization vision for discriminating objects while ignoring the variations in illumination caused by caustics. To explore this possibility, crabs (*Carcinus maenas*) and cuttlefish (*Sepia officinalis*), both of which have polarization vision, were presented with moving stimuli overlaid with caustics. Dynamic caustics inhibited the detection of an intensity-based stimulus but not when these stimuli were polarized. This study is the first to demonstrate that polarization vision reduces the negative impacts that dynamic illumination can have on visual perception.

## Teaser

Polarization vision helps to detect objects illuminated by flickering light in shallow water environments.

## INTRODUCTION

In many shallow water environments, downwelling light can be refracted from surface ripples to create a mesh of moving bands of illumination across the seafloor, known as water caustics (*1*). These are generated when concave and convex parts of the water’s surface refract and focus rays of light, creating areas of both converging and diverging incident light (*2*, *3*). This spatio-temporal variation of illumination is also described as caustic flicker, caustic networks, or wave lensing. Underwater, caustics are most visible when the incident light projects orthogonally onto the seafloor (*4*) with the spatial frequency of this pattern highest at around five times the wave’s crest-to-crest distance below the surface and will lessen with increasing depth, turbidity, and diffuseness of light (*3*, *5*).

Because water caustics create a continuously moving light environment, they can conceal the movement of animals, allowing prey to evade their predators or predators to hunt their prey while going unnoticed (*6*, *7*). By associating with visually noisy areas, individuals can exploit environments with reduced visibility by taking refuge within it, in turn influencing predator-prey interactions (*8*, *9*). This occurs because dynamic visual noise reduces an individual’s signal-to-noise ratio, whereby the caustic flicker interferes with the detection of visual information associated with the location of an animal (*10*).

Given the hindrances that dynamic visual noise can have on predator and prey visual systems, what physiological mechanisms may animals have evolved to mitigate these impacts? A popular theory put forward by Maximov (*11*) is that opponent channels in color vision may have evolved originally for this purpose. This is because the dynamic properties of caustics are largely achromatic and are therefore predicted to have only a small effect on the relative activity of spectral photoreceptors. Little evidence has been provided to support this hypothesis beyond demonstrating its feasibility in simulated visual systems (*12*). Furthermore, while Maximov focused his theories on color vision, there is also reason to believe that a similar hypothesis could explain the evolution of polarization vision underwater (*13*). Many invertebrates have evolved polarization vision and use it as an alternative to color for purposes such as navigation, habitat selection, communication, and predator/prey detection (*14*, *15*). Polarization sensitivity is known to increase salience in visual tasks by improving object-background contrast (*16*, *17*). It may also help with spotting transparent prey items [(*18*) but see (*19*)] or detecting objects that contain polarization reflections as part of communication strategies (*20*). Prey wavelength-based or intensity-based camouflage may also be exposed in polarization, such as in fish with silvery scales that mirror these modalities but not the polarization of light (*21*, *22*).

For Maximov’s theory to extend to the realm of polarization, it first needs to be demonstrated that dynamic caustics underwater show little or no modulation in polarization. While previous work investigating dynamic illumination of veiling light in the water column (looking horizontally through open water near the surface) recorded substantial modulation in both intensity and polarization (*23*), there has been no attempt to quantify the polarization content of dynamic caustics present on the seafloor. Given the relatively small fluctuations in refractive angle as sunlight travels through surface waters and given the low refractive indices between benthic objects and seawater, it seems unlikely that caustics on the seafloor would be accompanied by significant modulations in polarization. It remains unknown whether polarization vision in this context might be useful for mitigating the negative effects of dynamic illumination.

## RESULTS AND DISCUSSION

**Fig. 1.**
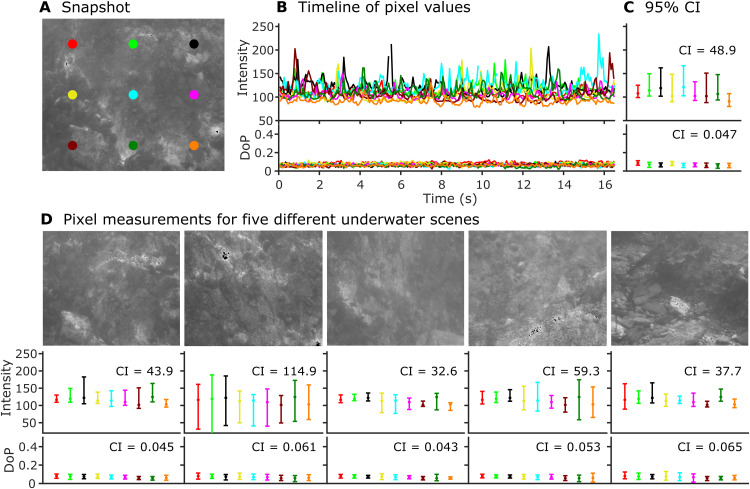
Intensity and polarization properties of natural caustics. (**A**) Intensity snapshot from a sequence filmed with a polarization camera (see movie S1 for full sequence). (**B**) Fluctuation of pixel values over time in intensity (top) and DoP (bottom). Each line is color-matched to its sample location [colored dots in (A)]. (**C**) Median and 95% CI for the data in (B). Overall mean CI indicated on each plot. (**D**) Same data as in (C) but collected from 100 frames (6.7 s) from each of five different scenes. High fluctuations in intensity, but not polarization, are observed on the seafloor in the presence of caustics.

To investigate whether caustic patterns in shallow waters show high degrees of noise in intensity but not polarization, we recorded video sequences of the shallow seafloor (~1 m depth) under sunny conditions off Mount Batten Beach, Plymouth, United Kingdom, using a polarization video camera. All video sequences showed large spatial and temporal fluctuations in intensity [mean 95% confidence intervals (CIs) of pixel values: 32.6 to 114.9 on the 256 increment 8-bit scale] but very small modulations in the degree of polarization (DoP; mean 95% CI: 0.043 to 0.065) (Fig. 1 and movie S1). General levels of polarization varied slightly depending on the overall reflected intensity of the substrate (fig. S2 and movie S2), likely driven by differences in the polarization of veiling light between the camera and the target but remain weakly polarized with little modulation over short time scales. Theoretically, therefore, polarization vision could help mitigate the impacts of visual noise under natural ecological conditions.

**Fig. 2.**
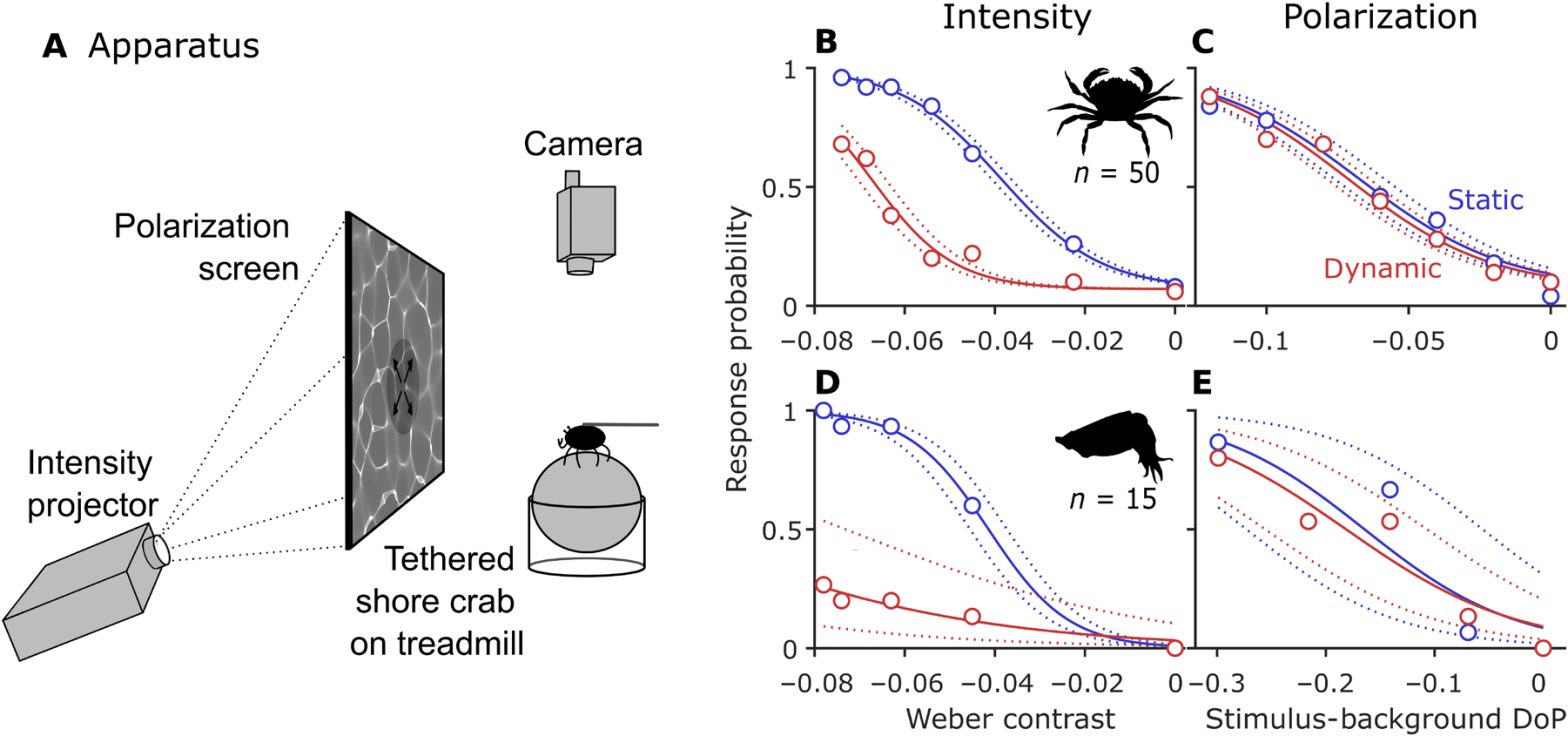
Response probability to visual stimuli. (**A**) Moving stimuli consisting of an expanding disc in intensity or polarization with an intensity-based caustic pattern overlaid were presented to animals in controlled conditions. Crab (**B** and **C**) and cuttlefish (**D** and **E**) responses were recorded to stimuli of varying contrast viewed either against static (plotted in blue) or dynamic (red) caustics. Circles indicate mean response probability for each stimulus contrast setting. Solid lines show the fitted sigmoid curve, and dotted lines indicate the 95% CI for the fitted curve. Polarization contrast is calculated as the stimulus DoP minus the background DoP.

To test whether animals use polarization vision to mitigate the problem of detecting objects in dynamically illuminated conditions, we used an established assay (*24*–*29*) to present shore crabs [*Carcinus maenas* (*30*)] and cuttlefish [*Sepia officinalis* (*30*)] with moving stimuli varying in intensity or polarization contrast overlaid with either stationary or moving caustics (Fig. 2A). Replicating natural caustics, these simulated caustics varied only in intensity but not polarization. The assay did not attempt to directly replicate natural conditions but could represent a scenario in which the target animal is approached by a predator viewed against a vertical or sloped background of rock or weed, such as may be found in large rockpools or shallow coastal waters. Dynamically moving caustics substantially impeded the ability of both species to detect an intensity-based moving stimulus (Fig. 2, B and D: chi-square comparison of glmm models with and without treatment as a factor in crabs; df = 1, χ^2^ = 121.7, *P* < 0.001; and cuttlefish: df = 1, χ^2^ = 67.1, *P* < 0.001). However, when the moving stimulus was presented in contrasts of polarization alone, the negative effects of the dynamic caustics on stimulus detection were abolished (Fig. 2, C and E: in crabs: df = 1, χ^2^ = 0.337, *P* = 0.56; and cuttlefish: df = 1, χ^2^ = 0.179, *P* = 0.67).

These experiments uncover evidence for a previously unknown function for polarization vision: the reduction of visual noise caused by fast flickering water caustics. Consistent with Maximov’s theory for the evolution of color vision underwater (*11*), these results suggest a powerful evolutionary driver for the development and maintenance of polarization vision in shallow aquatic environments. The benefits conveyed by mitigating dynamic visual noise are likely to have provided a substantial evolutionary advantage to animals foraging or hiding from predators in shallow clear waters of the ocean. This particularly applies to color-blind species such as the cuttlefish, whose polarization vision is the most acute among the animal kingdom studied so far (*28*) and appears to supersede the need for color.

Looking into the neural pathways involved in the use of polarization vision in this visual context may provide further understanding of how and why polarization sensitivity enhances object detection in visually noisy environments. Both crustaceans and cephalopods have two-channel polarization vision systems, in which half of their polarization-sensitive photoreceptors have microvilli (containing the visual pigments) aligned with the visual horizon and the other half oriented perpendicularly to the horizon (*31*, *32*). In crabs, the signal output from these photoreceptors connect with interneurons within the external plexiform layers of the lamina (*33*) to produce three output channels, (i) horizontal polarization, (ii) vertical polarization, and (iii) both channels combined to convey intensity information. The three channels terminate in the medulla, and it is theorized that signals from the two polarization channels project to a pathway independent of the intensity information, creating a parallel system for independently processing intensity and polarization of light (*17*, *34*, *35*). By being processed in parallel, the visual system is afforded access to a broader range of contrast information and enhances the ability to discriminate between objects and their background. This independent processing of polarization and intensity appears to be beneficial in water caustics; flickering illumination will prevent the binding of intensity-based features in the environment, but an object could still show polarization contrasts against the relatively stable unpolarized backdrop of the substrate despite unreliable intensity information. Polarization information, therefore, could remain effective for predators or prey performing different visual tasks in dynamically illuminated environments. Determining the effectiveness of polarization vision in this type of natural context should be the focus of future investigation.

Our study demonstrates a clear benefit for polarization vision in shallow waters, offering insights into how environmental conditions may have shaped the evolution of visual systems to mitigate the impacts of visual noise. Furthermore, both camera and digital processing technology are sufficiently advanced to replicate this in silico, offering opportunities to improve imaging systems for various forms of underwater platforms.

## MATERIALS AND METHODS

### Quantifying polarization in caustics

The polarization content of dynamic caustics in the natural environment was measured using a monochrome polarization camera (Blackfly S USB3 with Sony IMX250MZR sensor, Teledyne FLIR, Wilsonville, USA) mounted inside a custom-built underwater housing. Rather than recording frames with subpixels sensitive to red, green, and blue wavelengths (as in most standard cameras), frames consist of subpixels with four different orientations of polarization sensitivity: 0°, 45°, 90°, and 135°. Locations were selected on an ad hoc basis according to ease of access and only included footage from when the Sun was fully exposed. Pixel response was first linearized using a 0.8 gamma correction function. The four polarization channels were then processed to extract the first two stokes parameters using the formulae

$$S1=\frac{i90-i0}{i90+i0}$$ (1)

$$S2=\frac{i45-i135}{i45+i135}$$ (2)

where *i*0, *i*45, *i*90, and *i*135 refer to pixel values at each orientation of polarization sensitivity, and S1 and S2 are the first two stokes parameters. DoP was then calculated using the formula

$$\text{DoP}=\sqrt{S1^{2}+S2^{2}}$$ (3)

DoP values for each pixel were then assigned color values, where warmer colors represent higher levels of polarization. Any overexposed pixels were excluded from the analysis and assigned a “white” pixel in the false-color DoP images.

### Animals

*C. Maenas* of mixed sex were collected at low tide from Clevedon beach, United Kingdom (Global Positioning System: 51.43707, −2.86637), where they shelter under upper intertidal rocks and seaweed. The crab carapaces ranged from approximately 20 to 50 mm. They were collected in batches of 25 crabs and housed within individual plastic boxes in a shallow saltwater aquarium. Crabs were fed twice a week with defrosted mussels, cockles, or prawns. Temperature, salinity, and a natural light cycle were maintained for the duration of the experiment. Each crab was used in one experiment only and returned to the collection location within 1 week. Measurements were mostly carried out in the morning between 9 a.m. and midday.

*S. officinalis* of mixed sex were raised from eggs in aquaria at the Marine Biological Association, Plymouth, United Kingdom. In total, 25 individuals were tested ranging in size from 8 to 12 cm in mantle length. Each cuttlefish was fed a live shrimp three times a day or a single small crab once a day. *S. officinalis* were housed individually in fiberglass tanks (30 cm by 65 cm by 40 cm), which were cleaned twice per week. All tanks had a continuous circulation of water from a large flow-through system (supplied by fresh seawater from Plymouth sound) providing stable water quality throughout the experiment. Again, it was ensured that a naturally occurring light cycle was maintained for the duration of the experiment.

### Experimental apparatus

*C. maenas* were tethered above a spherical treadmill consisting of a Styrofoam ball (10 cm in diameter) suspended in a flow of air supplied from a compressed air tap into the bottom of a three-dimensional printed hemispherical cup (Fig. 2A and fig. S1A). Tethering involved gluing the “loop” part of a square of Velcro to the dorsal carapace of the crab using cyanoacrylate glue and then attaching this to a square of “hook” Velcro attached to a horizontally mounted metal rod. One of two custom-made stimulus screens was mounted against one open wall of the experimental arena constructed from a white fabric 50 cm by 50 cm by 50 cm Photo-Studio Cube. For intensity-only stimuli, two digital projectors (CP-WX3030WN, Hitachi, Tokyo, Japan), one presenting caustics and the other presenting predator-like expanding discs, were stacked on top of each other so that their images were overlaid onto a single semitransparent 30 cm by 38 cm rear projection screen (0.5 diffuser, Lee Filters, Andover, UK). For combined intensity and polarization stimuli, the predator-stimulus projector was replaced with a modified patterned vertical alignment type light-emitting diode (LCD) screen (1905fp, Dell, Round Rock, USA). Modification involved removing the LCD panel from the casing and stripping the frontmost polarizing filter from the screen’s surface, resulting in a panel that can modify the DoP of transmitted light in each pixel [see (*36*) for details]. A sheet of 0.5 diffuser (Lee Filters) was spray-glued to the innermost surface of the LCD panel, against which the intensity image of the digital projector was cast. This resulted in a combined display on which moving images could be produced varying in intensity and in horizontally oriented DoP [see (*17*) for further details].

On the first display, a simulated caustic flicker video generated using Caustics Generator Pro (movie S4; www.dualheights.se/caustics/) was played on loop (see table S1 for parameters used to create the pattern). The caustic patterns were either static or dynamic (moving) and were only ever presented in intensity contrast (i.e., there was no polarization component to the caustic flicker pattern). The static caustic flicker video had a refresh rate of 30 Hz (allowing for a reduced file size for the nonmoving stimulus video), and the dynamic caustic flicker had a refresh rate of 60 Hz. Both refresh rates are above the critical flicker fusion frequency of cuttlefish (*37*, *38*) and nocturnal or dark-adapted crabs (*39*, *40*) and so projected motion is likely to appear smooth to the visual systems of the test subjects, especially given the relatively low levels of illumination in the test arena compared with full daylight. The chosen spatial frequency of the pattern was determined using the 5-m-depth value on Caustics Generator Pro.

On the second display, a moving stimulus consisting of an expanding disc was animated using custom-written scripts in MATLAB (R2021a, MathWorks, Natick, USA) following established protocol from previous work on similar species (*24*–*26*, *28*). Discs followed a geometric expansion profile from a visual angle of 0° to ~23° (0 to 10.5 cm in diameter) against a plain background with a refresh rate of 30 Hz, expanding over a 3-s period for crabs and 1-s period for cuttlefish. The relative radiance of intensity-based expanding disc stimuli and the static caustic background was measured using a spectrophotometer (Flame, Ocean Insight, Largo, USA) coupled to an optic fiber (0.6 mm in diameter, 2 m in length; Ocean Insight). Radiance values in the 400- to 700-nm wavelength range were then used to calculate Weber contrast of the stimulus relative to the background. The same spectrophotometer coupled to a rotatable Glan-Thompson polarizer (Thorlabs, Newton, USA) was used to determine the DoP values of the stimulus and background using Eqs. 1 to 3 above. Audio beeps fed from the computer to a digital video camera recording crab behavior were used to synchronize the two data streams. These beeps were muted from external speakers so that the animals could not detect the sound.

The experimental apparatus used to study cuttlefish responses was similar to that used for crabs, with some exceptions relating to differences in natural history between the species. A rectangular aquarium tank (60 cm by 30 cm by 31 cm) was filled with 45 liters of circulating seawater and enclosed in a gazebo covered in black cloth to create a shaded environment (fig. S1C). Rocks were positioned in a V-shape at the back of the tank, which encouraged the cuttlefish to rest in a consistent position facing toward the stimulus screen (fig. S1D).

With the exception of the frontmost face of the aquarium, the inner surfaces were lined with white plastic glued to the glass to prevent distraction from reflections and to eliminate intensity artifacts caused by the internal reflection of polarized stimuli (*36*). For the first cuttlefish experiment involving intensity-only moving stimuli, a sheet of diffuser film (0.5, Lee Filters) was taped to the front surface of the tank to act as a rear projection screen. For the second cuttlefish experiment involving polarization-based moving stimuli, this diffuser was removed and the polarization screen was clipped onto the front surface of the tank (fig. S1E). Four sets of aquarium tanks were constructed side by side to enable a higher throughput of data collection (fig. S1F).

### Experimental protocol

Animals in each experiment were presented with a series of expanding disc stimuli viewed against either a dynamic or a static background of simulated intensity-based caustics. The order in which each series of contrasts was presented was fully randomized, and the order of dynamic and static backgrounds was alternated, so that half of the animals experienced a stimulus series on a dynamic background first, followed by static, and the opposite for the other half of the animals. For *C. maenas*, each crab was left to acclimatize on the treadmill for 3 min, before being shown one of the seven randomly ordered expanding disc stimuli with 1-min intervals between each stimulus (each crab spent approximately 20 min on the treadmill in total). After the experiment, the animal was removed from the treadmill and returned to its housing tank. Each crab was tested twice within each experiment (once for static and once for dynamic caustic conditions) in alternating order and with an interval of ~20 min between sessions for rehydration and recovery. Different crabs were used for each experiment, so 50 animals experienced the intensity-based moving stimuli, and 50 different animals experienced polarization-based moving stimuli. Trials were only started when a crab was actively walking on the treadmill, as this minimized the proportion of false negatives from already stationary crabs.

For *S. officinalis*, individual cuttlefish were transferred to the four experimental tanks using a net and bucket and were fed a live shrimp to help them settle and then were left to acclimatize for at least 4 hours before experiments began. At the start of experiments, the projector(s) were switched on and the first cuttlefish was left to acclimatize to the new light conditions for 10 min before being shown a series of five contrasts of moving stimuli in a random order with approximately 5-min intervals between each stimulus. Following this, the animals were left for 10 min to acclimatize to the second caustic treatment before experiencing the same five stimuli again in a different random order. As for the crabs, stimulus contrast was randomized within each series and the order of caustic treatment alternated between different cuttlefish. Different animals contributed to each experiment. However, because of lower overall numbers of animals available, five animals from the first intensity-based experiment had to be reused for the second polarization-based experiment. Trials were only initiated when the cuttlefish was still and facing toward the stimulus monitor.

### Response scoring

Videos of the animals were manually scored by an observer using a blind approach (i.e., no knowledge of stimulus contrast while scoring) to produce binary response data for each of the four experiments. Behaviors were only counted as a definite response when they occurred within the expansion phase of the stimulus. For *C. maenas*, positive responses included one or a combination of freezing or slowing to a stop, a clear increase or decrease in speed, tucking the legs and/or claws toward the body for protection, and extending the claws in an aggressive posture (movie S3). Fewer than five responses (of 50) were recorded to the zero-contrast stimulus in either crab experiment, implying a false-positive rate of <10%. For *S. officinalis*, a positive response included one or a combination of a very quick change of color across the whole or part of the body (including some very subtle changes), a sudden contraction of the body (jerk movement), or a sudden movement of the eyes toward the screen (movie S3). For cuttlefish, no responses were observed for the zero-contrast stimulus, implying a very low rate of false positives.

### Analysis

Similar to How *et al.* (*25*), the data were analyzed using a generalized linear mixed-model with one between-subjects factor, “caustic treatment” (static and dynamic), and one within-subjects factor, “stimulus contrast value” (5 to 7 Weber or polarization contrasts). The *glmer* function within the lme4 package was used in R 4.0.3 (CRAN, 2020), to compare both the effect of contrast and of caustic treatment on the crab and cuttlefish response using a binary distribution of data. The function *drop1* then compared models with and without each factor using a chi-square test statistic. Crab and cuttlefish identity was included as a random factor to account for the repeated measures design within each experiment.

### Ethics

All research was examined and approved by the University of Bristol’s Named Animal Care and Welfare Officer and the Animal Welfare and Ethics Review Board under document number UIN/21/061.

## Supplementary Materials

### This PDF file includes:

Table S1 Figs. S1 and S2 References

## Notes

View/request a protocol for this paper from *Bio-protocol*.

## References

[1] W. N. McFarland, E. R. Loew, Wave produced changes in underwater light and their relations to vision. Environ. Biol. Fishes 8, 173–184 (1983).

[2] J. A. Lock, J. H. Andrews, Optical caustics in natural phenomena. Am. J. Phys. 60, 397–407 (1992).

[3] Y. Y. Schechner, N. Karpel, "Attenuating natural flicker patterns" in *Oceans ‘04 MTS/IEEE Techno-Ocean ‘04 (IEEE Cat. No.04CH37600)*. (2004), vol. 3, pp. 1262–1268 Vol.1263.

[4] E. R. Loew, W. N. McFarland, "The underwater visual environment" in *The Visual System of Fish*, R. Douglas, M. Djamgoz, Eds. (Springer Netherlands, 1990), pp. 1–43.

[5] D. K. Lynch, W. C. Livingston, *Colour and light in nature*. (Cambridge University Press, 2001).

[6] S. R. Matchette, I. C. Cuthill, N. E. Scott-Samuel, Concealment in a dynamic world: Dappled light and caustics mask movement. Anim. Behav. 143, 51–57 (2018).

[7] J. R. Attwell, C. C. Ioannou, C. R. Reid, J. E. Herbert-Read, Fish avoid visually noisy environments where prey targeting is reduced. Am. Nat. 198, 421–432 (2021). 34403312 10.1086/715434

[8] S. R. Matchette, I. C. Cuthill, N. E. Scott-Samuel, Dappled light disrupts prey detection by masking movement. Anim. Behav. 155, 89–95 (2019).

[9] S. R. Matchette, I. C. Cuthill, K. L. Cheney, N. J. Marshall, N. E. Scott-Samuel, Underwater caustics disrupt prey detection by a reef fish. Proc. R. Soc. Lond. Ser. B Biol. Sci. 287, 20192453 (2020). 10.1098/rspb.2019.2453 PMC7209061 32228405

[10] J. A. M. Galloway, S. D. Green, M. Stevens, L. A. Kelley, Finding a signal hidden among noise: How can predators overcome camouflage strategies? Philos. Trans. R. Soc. Lond. B Biol. Sci. 375, 20190478 (2020). 32420842 10.1098/rstb.2019.0478 PMC7331011

[11] V. V. Maximov, Environmental factors which may have led to the appearance of colour vision. Philos. Trans. R. Soc. Lond. B Biol. Sci. 355, 1239–1242 (2000). 11079406 10.1098/rstb.2000.0675 PMC1692839

[12] P. G. Lovell, D. J. Tolhurst, C. A. Párraga, J. Troscianko, T. Troscianko, R. Baddeley, U. Leonards, Stability of the color-opponent signals under changes of illuminant in natural scenes. J. Opt. Soc. Am. A 22, 2060–2071 (2005). 10.1364/josaa.22.002060 16277277

[13] C. M. Talbot, J. Marshall, Polarization sensitivity and retinal topography of the striped pyjama squid (*Sepioloidea lineolata* – Quoy/Gaimard 1832). J. Exp. Biol. 213, 3371–3377 (2010). 20833931 10.1242/jeb.048165

[14] G. Horváth, D. Varjú, *Polarized light in animal vision*. (Springer-Verlag, 2004).

[15] G. Horváth, Ed., *Polarized light and polarization vision in animal sciences*, (Springer, 2014).

[16] M. J. How, J. H. Christy, S. E. Temple, J. M. Hemmi, N. J. Marshall, N. W. Roberts, Target detection is enhanced by polarization vision in a fiddler crab. Curr. Biol. 25, 3069–3073 (2015). 26585278 10.1016/j.cub.2015.09.073

[17] S. P. Smithers, N. W. Roberts, M. J. How, Parallel processing of polarization and intensity information in fiddler crab vision. Sci. Adv. 5, eaax3572 (2019). 31457103 10.1126/sciadv.aax3572 PMC6703871

[18] N. Shashar, R. T. Hanlon, A. de Petz, Polarization vision helps detect transparent prey. Nature 393, 222–223 (1998).9607759

[19] S. Johnsen, N. J. Marshall, E. A. Widder, Polarization sensitivity as a contrast enhancer in pelagic predators: Lessons from *in situ* polarization imaging of transparent zooplankton. Philos. Trans. R. Soc. Lond. B Biol. Sci. 366, 655–670 (2011). 21282169 10.1098/rstb.2010.0193 PMC3049004

[20] N. J. Marshall, N. W. Roberts, T. W. Cronin, "Polarisation signals" in *Polarized light and polarization vision in animal sciences,* G. Horvath, Ed. (Springer, 2014), pp. 407–442.

[21] T. M. Jordan, J. C. Partridge, N. W. Roberts, Non-polarizing broadband multilayer reflectors in fish. Nat. Photonics 6, 759–763 (2012). 23160173 10.1038/nphoton.2012.260 PMC3496938

[22] N. Shashar, R. Hagan, J. G. Boal, R. T. Hanlon, Cuttlefish use polarization sensitivity in predation on silvery fish. Vision Res. 40, 71–75 (2000). 10768043 10.1016/s0042-6989(99)00158-3

[23] S. Sabbah, N. Shashar, Underwater light polarization and radiance fluctuations induced by surface waves. Appl. Optics 45, 4726–4739 (2006). 10.1364/ao.45.004726 16799688

[24] V. Pignatelli, S. E. Temple, T. H. Chiou, N. W. Roberts, S. P. Collin, N. J. Marshall, Behavioural relevance of polarization sensitivity as a target detection mechanism in cephalopods and fishes. Philos. Trans. R. Soc. Lond. B Biol. Sci. 366, 734–741 (2011). 21282177 10.1098/rstb.2010.0204 PMC3049012

[25] M. J. How, V. Pignatelli, S. E. Temple, N. J. Marshall, J. M. Hemmi, High e-vector acuity in the polarisation vision system of the fiddler crab *Uca vomeris*. J. Exp. Biol. 215, 2128–2134 (2012). 22623201 10.1242/jeb.068544

[26] M. J. How, J. Christy, N. W. Roberts, N. J. Marshall, Null point of discrimination in crustacean polarisation vision. J. Exp. Biol. 217, 2462–2467 (2014). 24737768 10.1242/jeb.103457

[27] M. J. How, M. L. Porter, A. N. Radford, K. D. Feller, S. E. Temple, R. L. Caldwell, N. J. Marshall, T. W. Cronin, N. W. Roberts, Out of the blue: The evolution of horizontally polarized signals in *Haptosquilla* (Crustacea, Stomatopoda, Protosquillidae). J. Exp. Biol. 217, 3425–3431 (2014). 25104760 10.1242/jeb.107581

[28] S. E. Temple, V. Pignatelli, T. Cook, M. J. How, T. H. Chiou, N. W. Roberts, N. J. Marshall, High-resolution polarisation vision in a cuttlefish. Curr. Biol. 22, R121–R122 (2012). 22361145 10.1016/j.cub.2012.01.010

[29] S. E. Temple, M. J. How, S. B. Powell, V. Gruev, N. J. Marshall, N. W. Roberts, Thresholds of polarization vision in octopuses. J. Exp. Biol. 224, jeb240812 (2021). 10.1242/jeb.240812 PMC8077535 33602676

[30] C. Linnaeus, *Systema naturae*. (Laurentius Salvius, Stockholm, ed. 10th, 1758), vol. 1.

[31] M. F. Moody, J. R. Parriss, The discrimination of polarized light by Octopus: A behavioural and morphological study. Z. Vgl. Physiol. 44, 268–291 (1961).

[32] A. W. Snyder, Polarization sensitivity of individual retinula cells. J. Comp. Physiol. 83, 331–360 (1973).

[33] N. J. Strausfeld, D. R. Nässel, "Neuroarchitectures serving compound eyes of crustacea and insects" in *Handbook of sensory physiology,* H. Autrum, Ed. (Springer-Verlag, 1981), vol. VII/6B, pp. 1–593.

[34] R. M. Glantz, Polarization analysis in the crayfish visual system. J. Exp. Biol. 204, 2383–2390 (2001). 11511653

[35] M. J. How, N. J. Marshall, Polarization distance: A framework for modelling object detection by polarization vision systems. Proc. Royal Soc. B 281, 20131632 (2014). 10.1098/rspb.2013.1632 PMC3871304 24352940

[36] J. J. Foster, S. E. Temple, M. J. How, I. M. Daly, C. R. Sharkey, D. Wilby, N. W. Roberts, Polarisation vision: Overcoming challenges of working with a property of light we barely see. Naturwissenschaften 105, 27–27 (2018). 29589169 10.1007/s00114-018-1551-3 PMC5871655

[37] T. H. Bullock, B. U. Budelmann, Sensory evoked potentials in unanesthetized unrestrained cuttlefish: A new preparation for brain physiology in cephalopods. J. Comp. Physiol. A 168, 141–150 (1991). 2033566 10.1007/BF00217112

[38] L. Nelson, University of Plymouth, (2003).

[39] M. S. Grober, Luminescent flash avoidance in the Nnocturnal crab *Portunus xantusii*: II. Cardiac and visual responses to variations in simulated luminescent flashes. J. Exp. Biol. 148, 427–448 (1990).

[40] J. E. Layne, M. Wicklein, F. A. Dodge, R. B. Barlow, Prediction of maximum allowable retinal slip speed in the fiddler Crab,Uca pugilator. Biol. Bull. 193, 202–203 (1997). 9390385 10.1086/BBLv193n2p202

