# Supplementary material for: Polarization vision mitigates visual noise from flickering light underwater
Source: Sci Adv. 2022 Sep 9;8(36):eabq2770. doi: 10.1126/sciadv.abq2770 (PMC9462692; doi:10.1126/sciadv.abq2770)
Supplement: Supplementary file 1 — Table S1 Figs. S1 and S2 References [file sciadv.abq2770_sm.pdf]

Science Advances
AAAS

Supplementary Materials for

Polarization vision mitigates visual noise from flickering light underwater

Siân Vincent Venables *et al.*

Corresponding author: N. Justin Marshall, m.how@bristol.ac.uk

Sci. Adv. **8**, eabq2770 (2022)
DOI: 10.1126/sciadv.abq2770

The PDF file includes:

Table S1
Figs. S1 and S2
Legends for movies S1 to S4
References

Other Supplementary Material for this manuscript includes the following:

Movies S1 to S4

Supplementary table 1. Settings for Caustic Generator Pro for generating simulated caustics. Settings are identical for both static and dynamic treatments, but for static playback the first rendered frame was used only. A custom-written Matlab script was used to remove every 4th frame from the dynamic video sequence to increase the speed of movement.

**Parameter**	**Value**
Image size	1920 pixels
Number of frames in animation	200
Resolution	512
Depth	5 m
Intensity	0.05
Amplitude filter	34.44
Frequency filter	4.56
Time filter	55
Refraction spread	0.08
Light angle	0 °

Supplementary figures

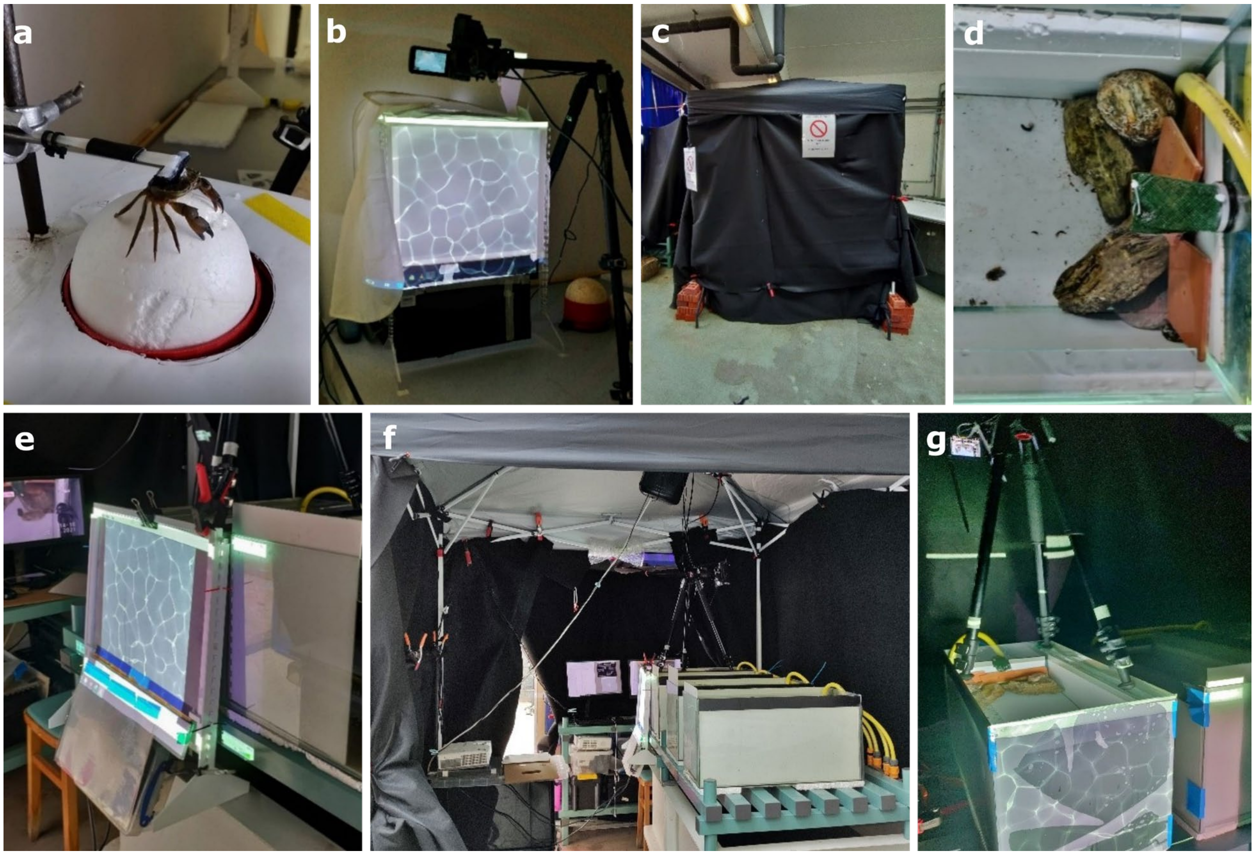

Supplementary figure 1. Photographs of the experimental apparatus for both *Carcinus maenas* and *Sepia officinalis.* (**A**) The spherical treadmill (consisting of a Styrofoam ball suspended in a flow of air) for clear observations of *C. maenas* responses to the presented stimulus. (**B**) The video camera and tripod positioned above the experimental area (photography tent) for recording responses of *C. maenas*. (**C**) The gazebo covered in black felt to enclose *S. officinalis* in darkness. (**D**) Rocks positioned in a V-shape to encourage *S. officinalis* to sit facing forwards towards the stimulus (in direction of arrow). (**E**) The polarization screen clipped onto the front surface of the tank for presenting the stimulus in polarized light. (**F**) The four sets of aquarium tanks constructed side-by-side to enable a higher throughput of data collection. (**G**) The video camera positioned on top of the tanks so that *S. officinalis* could be filmed from above. Photo credit: Siân Vincent Venebles, University of Bristol.

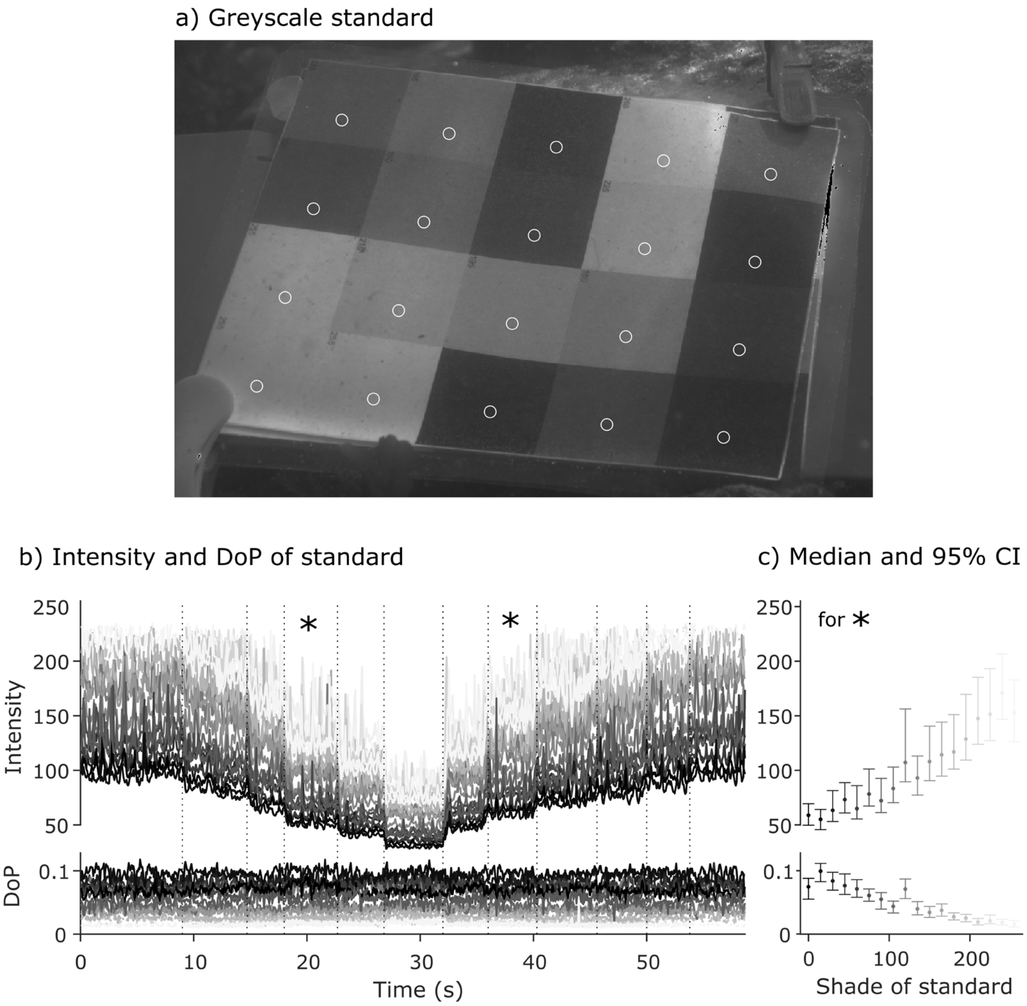

Supplementary figure 2. Polarization properties of caustics on a greyscale standard. a) Snapshot from polarization camera footage of the greyscale standard showing 20 randomly located squares varying in shade. White circles indicate sample points used to query pixel vales in b and c. b) Timeline showing pixel values in intensity (top) and degree of polarization (DoP, bottom) over time. Grey shade of each line corresponds to local shade of the greyscale standard. Vertical dotted lines indicate timepoint when camera aperture was adjusted. Stars indicate aperture settings that contribute to data in c. c) Median and 95% confidence interval for intensity (top) and DoP (bottom) pixel values within the starred time segments in b. See supplementary video 2.

Supplementary video captions

Supplementary video 1. Polarization properties of natural caustics. Video sequence captured with the polarization camera in shallow water showing intensity (left) and degree of polarization (DoP, right) properties of the dynamic caustics. The DoP image is false-colored according to the right-hand scale, which has the color range compressed into the bottom 50% of polarization values. White areas in the DoP image are areas of over-exposure.

Supplementary video 2. Polarization properties of greyscale standard. Video sequence captured with the polarization camera in shallow water showing intensity (left) and degree of polarization (DoP, right) properties of the dynamic caustics. The DoP image is false-colored according to the right-hand scale, which has the color range compressed into the bottom 50% of polarization values. White areas in the DoP image are areas of over-exposure.

Supplementary video 3. Example behavioral responses. Three crab and three cuttlefish example responses to expanding disc stimuli presented on background of caustics. Beeps are present on the original video and were used to link video timestamps with stimulus presentation. Inset left illustrates the expansion profile of the stimulus at the point of animal response.

Supplementary video 4. Static (left) and dynamic (right) caustics used for behavioral experiments. Videos are downsized by 50% for presentation due to file size.

REFERENCES AND NOTES

1. W. N. McFarland, E. R. Loew, Wave produced changes in underwater light and their relations to vision. *Environ. Biol. Fishes* **8**, 173–184 (1983).

2. J. A. Lock, J. H. Andrews, Optical caustics in natural phenomena. *Am. J. Phys.* **60**, 397–407 (1992).

3. Y. Y. Schechner, N. Karpel, "Attenuating natural flicker patterns" in *Oceans '04 MTS/IEEE Techno-Ocean '04 (IEEE Cat. No.04CH37600)*. (2004), vol. 3, pp. 1262–1268 Vol.1263.

4. E. R. Loew, W. N. McFarland, "The underwater visual environment" in *The Visual System of Fish*, R. Douglas, M. Djamgoz, Eds. (Springer Netherlands, 1990), pp. 1–43.

5. D. K. Lynch, W. C. Livingston, *Colour and light in nature*. (Cambridge University Press, 2001).

6. S. R. Matchette, I. C. Cuthill, N. E. Scott-Samuel, Concealment in a dynamic world: Dappled light and caustics mask movement. *Anim. Behav.* **143**, 51–57 (2018).

7. J. R. Attwell, C. C. Ioannou, C. R. Reid, J. E. Herbert-Read, Fish avoid visually noisy environments where prey targeting is reduced. *Am. Nat.* **198**, 421–432 (2021).

8. S. R. Matchette, I. C. Cuthill, N. E. Scott-Samuel, Dappled light disrupts prey detection by masking movement. *Anim. Behav.* **155**, 89–95 (2019).

9. S. R. Matchette, I. C. Cuthill, K. L. Cheney, N. J. Marshall, N. E. Scott-Samuel, Underwater caustics disrupt prey detection by a reef fish. *Proc. R. Soc. Lond. Ser. B Biol. Sci.* **287**, 20192453 (2020).

10. J. A. M. Galloway, S. D. Green, M. Stevens, L. A. Kelley, Finding a signal hidden among noise: How can predators overcome camouflage strategies? *Philos. Trans. R. Soc. Lond. B Biol. Sci.* **375**, 20190478 (2020).

11. V. V. Maximov, Environmental factors which may have led to the appearance of colour vision. *Philos. Trans. R. Soc. Lond. B Biol. Sci.* **355**, 1239–1242 (2000).

12. P. G. Lovell, D. J. Tolhurst, C. A. Párraga, J. Troscianko, T. Troscianko, R. Baddeley, U. Leonards, Stability of the color-opponent signals under changes of illuminant in natural scenes. *J. Opt. Soc. Am. A* **22**, 2060–2071 (2005).

13. C. M. Talbot, J. Marshall, Polarization sensitivity and retinal topography of the striped pyjama squid (*Sepioloidea lineolata* – Quoy/Gaimard 1832). *J. Exp. Biol.* **213**, 3371–3377 (2010).

14. G. Horváth, D. Varjú, *Polarized light in animal vision*. (Springer-Verlag, 2004).

15. G. Horváth, Ed., *Polarized light and polarization vision in animal sciences*, (Springer, 2014).

16. M. J. How, J. H. Christy, S. E. Temple, J. M. Hemmi, N. J. Marshall, N. W. Roberts, Target detection is enhanced by polarization vision in a fiddler crab. *Curr. Biol.* **25**, 3069–3073 (2015).

17. S. P. Smithers, N. W. Roberts, M. J. How, Parallel processing of polarization and intensity information in fiddler crab vision. *Sci. Adv*. **5**, eaax3572 (2019).

18. N. Shashar, R. T. Hanlon, A. de Petz, Polarization vision helps detect transparent prey. *Nature* **393**, 222–223 (1998).

19. S. Johnsen, N. J. Marshall, E. A. Widder, Polarization sensitivity as a contrast enhancer in pelagic predators: Lessons from *in situ* polarization imaging of transparent zooplankton. *Philos. Trans. R. Soc. Lond. B Biol. Sci.* **366**, 655–670 (2011).

20. N. J. Marshall, N. W. Roberts, T. W. Cronin, "Polarisation signals" in *Polarized light and polarization vision in animal sciences*, G. Horvath, Ed. (Springer, 2014), pp. 407–442.

21. T. M. Jordan, J. C. Partridge, N. W. Roberts, Non-polarizing broadband multilayer reflectors in fish. *Nat. Photonics* **6**, 759–763 (2012).

22. N. Shashar, R. Hagan, J. G. Boal, R. T. Hanlon, Cuttlefish use polarization sensitivity in predation on silvery fish. *Vision Res.* **40**, 71–75 (2000).

23. S. Sabbah, N. Shashar, Underwater light polarization and radiance fluctuations induced by surface waves. *Appl. Optics* **45**, 4726–4739 (2006).

24. V. Pignatelli, S. E. Temple, T. H. Chiou, N. W. Roberts, S. P. Collin, N. J. Marshall, Behavioural relevance of polarization sensitivity as a target detection mechanism in cephalopods and fishes. *Philos. Trans. R. Soc. Lond. B Biol. Sci.* **366**, 734–741 (2011).

25. M. J. How, V. Pignatelli, S. E. Temple, N. J. Marshall, J. M. Hemmi, High e-vector acuity in the polarisation vision system of the fiddler crab *Uca vomeris*. *J. Exp. Biol.* **215**, 2128–2134 (2012).

26. M. J. How, J. Christy, N. W. Roberts, N. J. Marshall, Null point of discrimination in crustacean polarisation vision. *J. Exp. Biol.* **217**, 2462–2467 (2014).

27. M. J. How, M. L. Porter, A. N. Radford, K. D. Feller, S. E. Temple, R. L. Caldwell, N. J. Marshall, T. W. Cronin, N. W. Roberts, Out of the blue: The evolution of horizontally polarized signals in *Haptosquilla* (Crustacea, Stomatopoda, Protosquillidae). *J. Exp. Biol.* **217**, 3425–3431 (2014).

28. S. E. Temple, V. Pignatelli, T. Cook, M. J. How, T. H. Chiou, N. W. Roberts, N. J. Marshall, High-resolution polarisation vision in a cuttlefish. *Curr. Biol.* **22**, R121–R122 (2012).

29. S. E. Temple, M. J. How, S. B. Powell, V. Gruev, N. J. Marshall, N. W. Roberts, Thresholds of polarization vision in octopuses. *J. Exp. Biol.* **224**, jeb240812 (2021).

30. C. Linnaeus, *Systema naturae*. (Laurentius Salvius, Stockholm, ed. 10th, 1758), vol. 1.

31. M. F. Moody, J. R. Parriss, The discrimination of polarized light by Octopus: A behavioural and morphological study. *Z. Vgl. Physiol.* **44**, 268–291 (1961).

32. A. W. Snyder, Polarization sensitivity of individual retinula cells. *J. Comp. Physiol.* **83**, 331–360 (1973).

33. N. J. Strausfeld, D. R. Nässel, "Neuroarchitectures serving compound eyes of crustacea and insects" in *Handbook of sensory physiology,* H. Autrum, Ed. (Springer-Verlag, 1981), vol. VII/6B, pp. 1–593.

34. R. M. Glantz, Polarization analysis in the crayfish visual system. *J. Exp. Biol.* **204**, 2383–2390 (2001).

35. M. J. How, N. J. Marshall, Polarization distance: A framework for modelling object detection by polarization vision systems. *Proc. Royal Soc. B* **281**, 20131632 (2014).

36. J. J. Foster, S. E. Temple, M. J. How, I. M. Daly, C. R. Sharkey, D. Wilby, N. W. Roberts, Polarisation vision: Overcoming challenges of working with a property of light we barely see. *Naturwissenschaften* **105**, 27–27 (2018).

37. T. H. Bullock, B. U. Budelmann, Sensory evoked potentials in unanesthetized unrestrained cuttlefish: A new preparation for brain physiology in cephalopods. *J. Comp. Physiol. A* **168**, 141–150 (1991).

38. L. Nelson, University of Plymouth, (2003).

39. M. S. Grober, Luminescent flash avoidance in the Nnocturnal crab *Portunus xantusii*: II. Cardiac and visual responses to variations in simulated luminescent flashes. *J. Exp. Biol.* **148**, 427–448 (1990).

40. J. E. Layne, M. Wicklein, F. A. Dodge, R. B. Barlow, Prediction of maximum allowable retinal slip speed in the fiddler Crab,Uca pugilator. *Biol. Bull.* **193**, 202–203 (1997).
